# Characterization of Fruit Vinegars via Bioactive and Organic Acid Profile Using Chemometrics

**DOI:** 10.3390/foods12203769

**Published:** 2023-10-13

**Authors:** Elif Yildiz

**Affiliations:** Food Processing Department, Keles Vocational School, Bursa Uludag University, 16740 Bursa, Türkiye; elifyildiz@uludag.edu.tr

**Keywords:** vinegar, organic acids, phenolic compounds, antioxidant capacity, bioaccessibility

## Abstract

Vinegar has been known as a traditional remedy since ancient times. In addition to being used as a flavoring and aroma enhancer in world cuisines, it has attracted more and more attention due to its bioactive potential and health properties. Although the most common use is apple cider vinegar together with grape vinegar, vinegar produced from red fruits has come to the fore due to their health purposes. Rosehip, pomegranate, fig, guelder-rose, blackberry, raspberry, and blueberry vinegars were evaluated regarding the organic acid content, phenolic compound content, and bioactive potential to assess their health potential and associated contents. Acetic acid, citric acid, succinic acid, and malic acid were determined as prominent organic acids in the vinegar samples. In contrast, gallic acid, vanillic acid, protocatechuic acid, and ferulic acid were dominant regarding phenolic compounds. Raspberry, guelder-rose, and pomegranate vinegars came forth regarding their bioactive content and potential. The discriminative parameters of the vinegar samples were pH, total acidity, dL-isocitric acid, gallic acid, and hydroxybenzoic acid. Fruit vinegars were determined to have a notable bioactive content compared to apple and grape vinegars. The use of these vulnerable bioactive materials in vinegar fermentation could provide an effective way for nutrition and raw material resourcing.

## 1. Introduction

Today, due to the understanding of the importance of nutrition, the effects of food on overall health and wellness have come to the fore. In particular, studies on the bioactive potential of foods have increased, consumer habits have changed, and importance has been given to healthy and health-supportive foods. Most notably, fermented food products have gained a remarkable importance.

Fermentation is a very valuable bioprocess that improves the nutritional and sensorial properties of food and also extends their storage and usage periods. The biochemical steps that occur during the fermentation process alter the nutritive and antinutritive components and improve the nutritional qualifications. These are the bioactivity, bioaccessibility, and digestibility of the food content. Microbial enzymes break the cell wall matrix of the plants and facilitate the release of the bioactive content extraction. These enzymes are also crucial participants in the chemical reactions responsible for the decomposition of prevalent compounds leading to the formation of brand-new metabolites. Also, bioactive compounds with a high molecular weight are broken down into low molecular weight ones [1,2]. Additionally, fermentation helps to create new food products and allows for the evaluation of perishable fruits, vegetables, and plants that are only available for a short time.

Vinegar, a fermented product that has been consumed for many years, is added to foods to improve the taste. In the 17th century, it began to be used medicinally by Europeans. It has been included in syrups and antiseptics as an antimicrobial agent. Today, in addition to its widespread use as a dressing in foods such as salads, its use is increasing day-by-day due to its positive effects on health [2]. Vinegar production is a two-step fermentation process. Fermentable carbohydrates are first converted into ethanol by yeasts, often of the *Saccharomyces* genus. Then, the ethanol is oxidized by bacteria, typically of the *Acetobacter* genus. Fermentation is a crucial process in vinegar production, which involves the chemical and microbiological modification of several organic acids, phenolic compounds, volatile compounds, vitamins, minerals, and other substances [3]. According to recent studies, vinegar possesses antioxidant, anti-inflammatory, antibacterial, and anticarcinogenic effects [4]. The bioactive content of vinegar, notably attributed to its acidic and bioactive content, has the ability to have health benefits [3]. Vinegar has a high concentration of organic acids, which are both the primary contributor to vinegar’s flavor and an essential factor in determining the product’s overall quality. The composition of organic acids found in vinegars plays a significant role with regard to not only the flavor but also the nutritional value and bioavailability. Also, the antimicrobial effect, particularly acetic acid, in vinegars is attributed to have the ability to permeate the cell membranes, leading to the breakdown being a naturally produced antimicrobial agent [4].

Fruits possess a wealth of bioactive substances that are crucial in conferring health advantages. These include phytochemicals, dietary fibers, a great prevalence of minerals in trace amounts as well as various bioactive potentials promoting overall well-being [1]. Additionally, phenolic compounds such as hydroxycinnamic acids and their derivatives, namely *p*-coumaric, ferulic, caffeic, and sinapic acids, exhibit a wide distribution in various fruits [5].

The use of fruit vinegars, namely red fruit vinegars, has become increasingly prominent in the field of nutrition as an approach to boost the effectiveness and benefits of these substances. As raw materials of vinegar fermentation, they are all rich in sugars (glucose, fructose) and contain a high content of dietary fiber (cellulose, hemicellulose, pectin), organic acids (citric acid, malic acid, tartaric, oxalic, and fumaric acid, etc.), and a great prevalence of minerals in trace amounts [6]. The presence of bioactive compounds within the fruit is complemented by the significant influence of the fermentation process [1]. Again, the health-promoting bioactive potential is closely associated with its bioactive compound content [3]. For the prediction of this bioactive potential, antioxidant capacity assays reflect the potential of the overall content. The evaluation of the bioaccessible phenolic content is also more realistic for providing the mimic extraction conditions of gastrointestinal digestion in terms of the health-related potential of contents.

In this study, the organic acid and phenolic compound profiles of red fruit vinegars which were prepared by rosehip (*Rosa canina*), pomegranate (*Punica granatum* L.), fig (*Ficus carica*), guelder-rose (*Viburnum opulus*), blackberry (*Rubus fruticosus* L.), raspberry (*Rubus idaeus*), and blueberry (*Vaccinium corymbosum* L.) were evaluated. Apple and grape vinegar, which are the most widely produced and easily accessible to consumers, were also included in the samples. Furthermore, the effect of the bioactive compound content on in vitro bioactivity and bioaccessibility of vinegars was evaluated in this concern.

## 2. Materials and Methods

### 2.1. Materials

Vinegar samples were obtained from an artisan vinegar producer (Vinegral, Bursa, Türkiye). Apple, grape, rosehip, pomegranate, fig, guelder-rose, blackberry, raspberry, and blueberry fruits were obtained from local organic farmers (Bursa, Türkiye) in 2022.

### 2.2. Chemicals

All organic acid and phenolic compound standards were obtained from Sigma-Aldrich (St. Louis, MO, USA). Chemicals used in physicochemical, antioxidant capacity, total phenolic content, and total anthocyanin analysis were provided by Merck (Darmstadt, Germany).

### 2.3. Methods

#### 2.3.1. Vinegar Production

Fermentation was produced according to the traditional fermentation method [7]. Fruits were crushed, and 3.5 kg of fruit was gathered with 10 L of sterilized drinking water. The content was left for alcoholic fermentation at room temperature in an airtight way, and kept away from sunlight. After completing the alcoholic fermentation, pre-produced vinegar and mother vinegar (250 mL) were added in equal amounts to each container. The content was left for acetic acid fermentation at room temperature, and the containers were covered with double-layer cheesecloth. After acetic acid fermentation, vinegar samples were kept in hermetic glass bottles at room temperature (20 ± 2 °C, in the dark) till analysis.

#### 2.3.2. Physicochemical Analyses

The total acidity of samples was determined according to AOAC [8] (Method No: 942.15) and expressed as acetic acid equivalent. Samples’ pH values were evaluated according to AOAC [9] (Method No: 981.12) by pH-meter (S220-K Seven Compact, Mettler Toledo, Milano, Italy). The measurements were conducted in triplicate, the results were given as mean ± standard deviation (SD).

#### 2.3.3. Organic Acid Determination

The organic acid contents of vinegar samples were defined by HPLC (1260 Infinity LC model, Agilent Technologies, Santa Clara, CA, USA) in terms of benzoic acid, L-ascorbic acid, acetic acid, adipic acid, benzoic acid, butyric acid, citric acid, isobutyric acid, formic acid, fumaric acid, L-(+)-lactic acid, dL-isocitric acid, (−)-quinic acid, maleic acid, malonic acid, D-(−)-tartaric acid, D-(+)-malic acid, oxalic acid, phytic acid, propionic acid, succinic acid, shikimic acid (Sigma-Aldrich Co., St. Louis, MO, USA). The methodology was structured according to Coelho et al. [10] with slight modifications.

For the extraction, 15 mL of sample and 15 mL ultra-pure water were mixed and shaken in a water bath (250 rpm, 25 °C, 30 min) and then centrifuged (1000 rpm, 15 °C, 10 min; 3 K 30, Sigma, Roedermark, Germany). The supernatant was collected, filtered through a 0.45 μm disc syringe filter, and vialed for injection.

**Figure 1:**
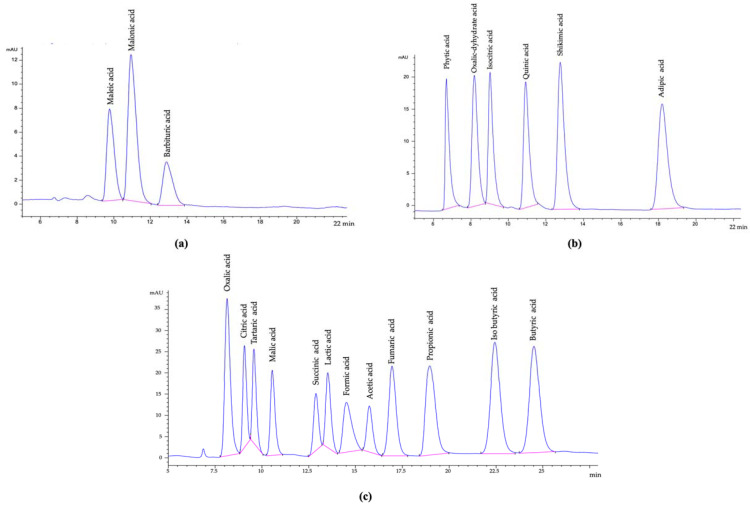
(**a**–**c**) Chromatograms of the organic acid standards (Signal 210/4 nm Ref; 400/100 nm).

HPLC was equipped with DAD (diode array detector) (1260, G1315C model, Agilent, Santa Clara, CA, USA), and an ion-exchange column was used (300 × 7.7 mm, 8 µm; Hi-Plex H, AGPL1170-6830, Agilent, Santa Clara, CA, USA). The mobile phase was 0.02 N H_2_SO_4_ with 0.6 mL/min flow rate at 50 °C with 36.5 bar detector pressure for an isocratic operational system with 10 μL sample injection volume. The spectra were recorded at 210 nm (Signal 210/4 nm Ref; 400/100 nm; Figure 1a–c). Performance parameters of the analytical method are given in Table 1 for organic acid determination methodology. The measurements were conducted in triplicate, the results were given as mean ± standard deviation (SD).

#### 2.3.4. Phenolic Compound Determination

The individual phenolic compounds of vinegar samples were defined by HPLC (1260 Infinity LC model, Agilent Technologies, Santa Clara, CA, USA) in terms of ascorbic acid, gallic acid, protocatechuic acid, catechin, hydroxybenzoic acid, vanillic acid, gentisic acid, *p*-coumaric acid, *o*-coumaric acid, coumarin, rutin, ferulic acid, naringin, neohesperidin, resveratrol, quercetin, trans-cinnamic, hesperidin, alizarin, and flavone (Sigma-Aldrich Co., St. Louis, MO, USA). The methodology was structured according to Selli [11] with slight modifications.

An amount of 15 mL of the vinegar sample was mixed with 15 mL ultra-pure water and 30 mL ethyl acetate; then, the mixture was vortexed vigorously and kept in the dark at room temperature (20 ± 2 °C, in the dark) for 96 h. At the end of the time, two phases were obtained in the mixture, and the upper phase was taken and treated by a rotary evaporator for 10 min (45 °C, 250 rpm). An amount of 2 mL of methanol was added to the residue, vortexed vigorously, and filtered into the vial through a 22 μm.

**Figure 2:**
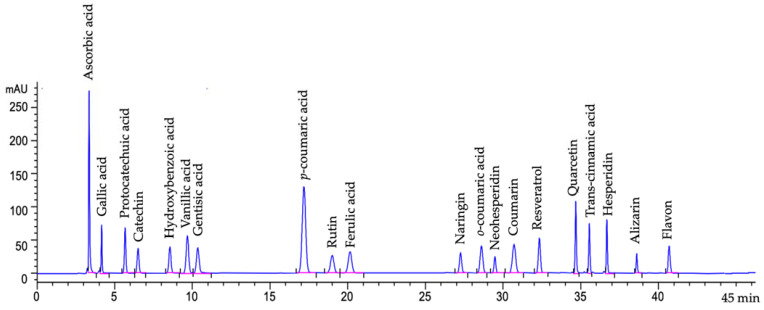
Chromatogram of the phenolic compound acid standards (Signal 300/200 nm Ref; 500/100 nm).

HPLC was equipped with a diode array detector (DAD) (1260, G1315C model, Agilent, Santa Clara, CA, USA), and a C_18_ column (250 × 4.6 mm, 5 μm; ACE Generix^®^, Advanced Chromatography Technologies, Aberdeen, UK) was used for the separation. Mobile phase-A was phosphoric acid solution (0.1%, *v*/*v*), and -B was acetonitrile. The procedure of gradient elution was set as 0 min, 17% (B); 7 min, 15% (B); 20 min, 20% (B); 24 min, 25% (B); 28 min, 30% (B); 30 min, 40% (B); 32 min, 50% (B); 36 min, 70% (B); and 40 min, 17% (B). The injection volume was 10 μL, and the flow rate was 0.8 mL/min at 30 °C with 36.5 bar detector pressure. The spectra were recorded at 300 nm (Signal 300/200 nm ref; 500/100 nm). Chromatogram of the phenolic compound acid standards are given in Figure 2. The measurements were conducted in triplicate, the results were given as mean ± standard deviation (SD).

#### 2.3.5. Antioxidant Capacity and Total Phenolic Content

The bioactive potential of samples was evaluated via DPPH antioxidant capacity (AC) assay and total phenolic content (Folin Ciocalteu’s method) analysis. Vinegars were evaluated as three different phenolic fractions: extractable phenolic fraction (EPF), hydrolysable phenolic fraction (HPF), and bioaccessible phenolic fraction (BPF).

##### Extraction Procedure

EPF and HPF extractions were obtained according to Vitali et al. [12]. An amount of 2 mL vinegar was mixed with 20 mL of HCl/methanol/H_2_O (1:80:10, *v*/*v*), then shaken in a water bath (250 rpm, 20 °C, 2 h). Afterward, the mixture was centrifuged (10 min, 4 °C, 3500 rpm; 3 K 30, Sigma, Roedermark, Germany), and the supernatant was kept as EPF. For HPF, 20 mL H_2_SO_4_/methanol (1:10 *v*/*v*) was added to the residue, then shaken in a water bath (20 h, 85 °C, 250 rpm). Finally, the mixture was centrifuged (10 min, 4 °C, 3500 rpm; 3 K 30, Sigma, Roedermark, Germany), and the supernatant was kept as HPF. BFF was obtained according to Bouayed et al. [13] by in vitro enzymatic digestion extraction. An amount of 2 mL vinegar was treated to the pepsin enzyme (40 mg/mL in 0.1 M HCl, pH: 2) and incubated (37 °C, 2 h) in a shaking water bath (250 rpm). Afterward, the porcine pancreatic enzyme (2 mg/mL) and porcine bile (12 mg/mL) were added to the mixture (pH: 7.2) and incubated (37 °C, 2 h) in a shaking water bath (250 rpm) again. Finally, the mixture was centrifuged (10 min, 15 °C, 3500 rpm; 3 K 30, Sigma, Roedermark, Germany), and the supernatant was kept as BPF. All extracts were stored at −20 °C until the analyses.

##### Determination of Antioxidant Capacity

The antioxidant capacity (AC) of vinegar samples was evaluated in terms of the DPPH (2.2diphenyl-1-picrylhydrazyl) AC assay. The DPPH method was conducted according to Brand-Williams et al. [14]. Sample extracts were kept in the dark with 6 × 10^−5^ M DPPH solution for 30 min. Their absorbance was determined spectrophotometrically (UV 1208, Shimadzu, Japan) at 515 nm. Methanol was used as the blank, Trolox^®^ (0.02–0.08 µmol) was used as the standard, and Trolox^®^-based calibration curve of y = 3246.2x + 0.7181 (R^2^ = 0.9941) was utilized in calculations. The results were expressed as µmol Trolox^®^ equivalents (TE) per mL sample. All the determinations were carried out in triplicate, and all results are given as mean ± standard deviation.

##### Determination of Total Phenolic Content

The total phenolic content (TPC) of vinegar samples was determined via the procedures of Apak et al. [15]. Lowry A solution was obtained by dissolving 2% Na_2_CO_3_ in 0.1 mol/L NaOH; Lowry B solution was obtained by dissolving 0.5% CuSO_4_ in 1% NaKC_4_H_4_O_6_ solution. Then, Lowry A and Lowry B solutions were mixed homogeneously at a ratio of 50:1 (*v*/*v*) for the Lowry C solution. Sample extracts were mixed with Lowry C solution and kept in the dark for 10 min. Afterward, Folin Ciocalteu’s reagent was added, and the mixture was kept in the dark for 30 min. The absorbance was measured spectrophotometrically (Shimadzu UV 1208, Japan) at 750 nm. Distilled water was used as the blank, gallic acid (10–500 mg/L) was used as the standard, and a gallic acid-based calibration curve of y = 0.346x − 0.0182 (R^2^ = 0.9997) was utilized in calculations. The obtained results were expressed as mg gallic acid equivalent (GAE) per mL sample, measurements were conducted in triplicate, and all results are given as mean ± standard deviation (SD).

##### Bioaccessibility %

The percentage of bioaccessibility of vinegar samples was calculated using the following equation [16] by the obtained results from the AC and TPC analysis.

$$\mathrm{Bioaccessibility}\;\%=\frac{BPF}{EPF+HPF}\;\times 100$$

#### 2.3.6. Determination of Total Anthocyanin Content

The total anthocyanin content (TAC) of vinegar samples was determined via the spectrophotometric pH-differentiation method that was proposed by Lee et al. [17]. An amount of 3 mL of vinegar sample was mixed with 12 mL distilled water. Afterward, 1 mL of the sample solution was separately mixed with 4 mL of pH: 1.0 and 4 mL of pH: 4.5 buffer solutions. The absorbances were measured spectrophotometrically (Shimadzu 1208, Kyoto, Japan) for 510 nm and 700 nm. The measurements were conducted in triplicate, the results were given as cyanidin-3-glucoside (C3G) equivalent, and all results are given as mean ± standard deviation (SD).

#### 2.3.7. Statistical Analysis

The results of the vinegar samples were evaluated statistically by using variance analysis with statistical software (Statistical Discovery from SAS 2005. Institute Inc., Cary, NC, USA). The LSD (least significant differences) test was used to determine the statistical difference between the means. Additionally, the linear correlation between the datasets was determined. Principal component analysis (PCA) models were subsequently employed to classify observations of unknown class origin by forecasting the data into each PCA class model while considering a 95% confidence range (Pirouette^®^ software, v4.5, Infometrix Inc., Bothell, WA, USA). The SIMCA methodology is a supervised classification method based on PCA. The aim is to construct a unique PCA model for every identified data category. The performance of the SIMCA models was evaluated using various metrics, including misclassification, interclass class distances (ICD), discriminating power plots, class projections, specificity, sensitivity, and accuracy.

## 3. Results and Discussion

### 3.1. pH and Total Titratable Acidity

**Figure 3:**
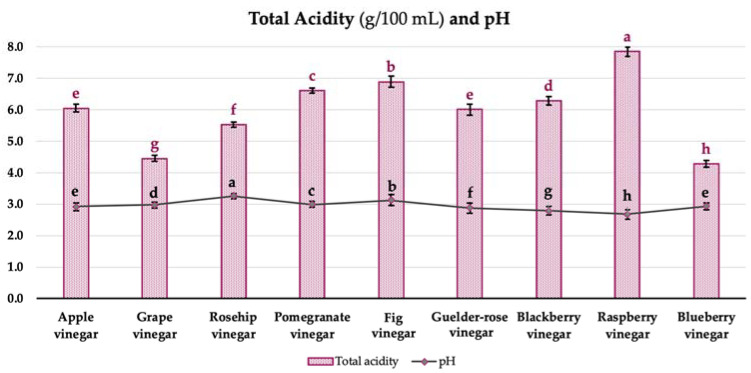
Total acidity and pH values of vinegar samples. Values represented by bar graphs are given as mean ± SD, letters in red (a–h) represent statistical differences between samples for total acidity values (acetic acid equivalent, *p* < 0.05), letters in black (a–h) represent statistical differences between samples for pH values (*p* < 0.05).

The general acceptability and organoleptic quality of fruit vinegars are greatly influenced by the organic acid content [18]. The pH and total acidity values of the vinegar samples are given in Figure 3. The total acidity values of the samples were changed between 4.28 ± 0.07 and 7.84 ± 0.16 g/100 mL (acetic acid equivalent), while the pH values were varied between 2.67 ± 0.07 and 3.26 ± 0.14 (Figure 3, *p* < 0.05). The total acidity of the samples was found to conform with the international standards [19], as indicated by 4% (*w/v*). 

Budak [20] determined the total acidity of apple, grape, and pomegranate vinegars, respectively, as 5.51–7.38%, 8.59–12.29%, and 5.72%, and the pH values as 2.87–3.21, 2.87–2.90, and 3.08. Increasing the sugar content of fruit (the raw material) might provide a relatively higher acidity development in vinegars. Additionally, Sengun et al. [21] evaluated the commercially available fruit vinegars. The results revealed that the pH values ranged from 3.22 to 3.85, while the total acidity levels varied between 1.11 and 5.61% (acetic acid equivalent). Furthermore, Bakir et al. [22] expressed the pH and total acidity levels observed in fruit vinegar as within the range of 2.8 to 3.9 and 0.7 to 6.6% (acetic acid equivalent), respectively.

The total acidity values of the samples were relatively higher compared to the commercial vinegars due to traditional fermentation by the organic raw materials and being an artisan product. Additionally, a higher sugar content in the raw material might provide further fermentation and the relatively higher acidity development in vinegars.

### 3.2. Organic Acids

Known for its antimicrobial properties, the potential of vinegar is dedicated to its organic acid content, especially acetic acid. In this study, vinegar samples were evaluated in terms of 21 different organic acids. Acetic acid was the most abundant as an expected result of the acetic acid fermentation of vinegar production. Its content was changed from 29,968.43 ± 320.06 mg/L (grape vinegar) to 60,592.87 ± 318.75 mg/L (raspberry vinegar). Acetic acid was followed by citric acid (8723.06 ± 25.15 mg/L), succinic acid (6045.12 ± 11.05 mg/L), malic acid (4852.44 ± 12.06 mg/L), adipic acid (3766.3 ± 15.34 mg/L), and lactic acid (1547.89 ± 21.45 mg/L), respectively. Among all the analyzed vinegar samples, raspberry (63,346.93 ± 69.50 mg/L), guelder-rose (61,418.06 ± 43.08 mg/L), and pomegranate (56,961.83 ± 84.13 mg/L) vinegars exhibited a notably high level of organic acid content. For the determined organic acid diversity, apple vinegar came to the fore via the detection of 16 different organic acids (Table 2). Despite containing seven different organic acids, pomegranate vinegar had a higher content of citric acid (5335.26 ± 42.07 mg/L), isocitric acid (967.35 ± 1.07 mg/L), and malic acid (3971.76 ± 39.36 mg/L) compared to other vinegars. Raspberry vinegar was noted for the highest organic acid content (63,346.93 ± 69.50 mg/L). Following acetic acid, predominantly citric acid (1904.66 ± 1.75 mg/L), dL-isocitric acid (325.49 ± 0.30 mg/L), and succinic acid (304.51 ± 2.69 mg/L) were involved in its organic acid content. Blueberry vinegar exhibited a higher content of malonic acid (123.01 ± 0.15 mg/L) and quinic acid (244.75 ± 0.82 mg/L), while guelder-rose vinegar demonstrated a greater abundance of succinic acid (1902.47 ± 1.67 mg/L) and shikimic acid (17.67 ± 0.02 mg/L) at a statistically significant level (*p* < 0.05) compared to other vinegars. Additionally, neither formic nor phytic acid was identified in the samples.

Generally, vinegars are determined to contain acetic acid, citric acid, formic acid, lactic acid, malic acid, succinic acid, and tartaric acid [23]. Ousaaid et al. [3] indicated the succinic acid presence was 3.92 to 6.43%, as the second-most abundant after acetic acid, while oxalic and malic acids had the lowest quantities within the scope of their comprehensive review of fruit vinegars. Liu et al. [24] evaluated 23 fruit vinegar samples and indicated tartaric acid, malic acid, lactic acid, citric acid, and succinic acid as the most widely distributed organic acids after acetic acid. Song et al. [25] determined acetic acid (59.11 ± 8.48 g/L), oxalic acid (3.38 ± 1.77 g/L), citric acid (0.06 ± 0.01 g/L), lactic acid (0.77 ± 0.08 g/L), and malic acid (0.50 ± 0.10 g/L) in raspberry vinegar samples.

Fruits are comprised of metabolically active living tissues that undergo continuous changes in their composition. The specific ratio and extent of these changes are contingent upon the fruit’s physiological role and state of maturity. They possess a higher concentration of reducing sugars compared to sucrose. Together with sugars, tissues also possess sugar alcohols and sugar acids [7]. Tropical and sub-tropical fruits, especially, include higher amounts of glucose and fructose (pomegranate, persimmon, etc.) as a combination of the two sugars higher than 10%. Only grapes are conceivably known to have more than 10% as a temperate fruit [7]. Organic acids are produced by fermentation due to processes such as microbial activity, biochemical metabolism, and hydrolysis. These mechanisms lead to the degradation of proteins and carbohydrates through small sugars, peptides, and amino acids. In order to undergo subsequent metabolic processes, carbohydrates are converted into pyruvic acid through intermediate steps via the Embden Meyerhof Parnas pathway and the hexose monophosphate pathway, which further produce organic acids [4,7]. Via the aerobic Krebs cycle, the acetic acid bacteria oxidize ethanol, sugar alcohols carbohydrates, to organic acids, aldehydes, and ketones, etc. In oxidative fermentation, they transform glucose into gluconic acid via glucono-delta-lactone and ethanol to acetic acid via the pyruvate and citric acid cycle [26]. The tricarboxylic acid cycle, the ultimate metabolic route of three key nutrients (carbohydrate, lipid, amino acid), produces energy from vinegar’s organic acids such as malic, citric, succinic, and lactic acids [4]. The ratios to apple and grape vinegar, which draw attention due to frequent use, studies have shown that acetic acid constitutes most of the organic acid content (accounting for 79.9% and 84.2% in the grape vinegar and apple vinegar, respectively) [27]. For this reason, the variable content and amount of sugars are mainly responsible for the produced organic acid prevalence of the vinegar samples (Table 2) as a primary substrate of vinegar fermentation. Along similar lines, Ren et al. [28] evaluated fruit and cereal vinegar, and they stated that fruit vinegars are determined to have a more complex organic acid composition and content compared to cereal ones. This is also evidence for the organic acid diversity of fruit vinegars.

In addition to the chemical composition present in utilized fruits in vinegar fermentation, the alcoholic and acetic acid fermentation processes are very effective on the final vinegar content. Kim et al. [29] explained the metabolite profile of tomato vinegar during fermentation in terms of alcoholic and acetic acid fermentation. They observed a consistent upward trend in oxalic acid, malonic acid, glutamic acid, linoleic acid, and glutaric acid concentrations throughout both of the alcoholic fermentation and acetic acid fermentation processes. It is noteworthy that the levels of malic acid, galactaric acid, tartaric acid, and glycerol monostearate increased after alcoholic fermentation and a decrease was detected after acetic acid fermentation. Also, they concluded that the optimization of strain selection for both alcoholic fermentation and acetic acid fermentation might lead to the maximization of particular metabolite synthesis. Additionally, Ozdemir and Budak [30] evaluated rose vinegar fermentation and determined that citric acid increased significantly following acetic acid in acetic acid fermentation. Regarding the levels of malic acid and oxalic acid, it is noteworthy that the rise in the oxalic acid content was significant. According to Velioglu [23], lactic acid bacteria were determined to have the potential to produce succinic acid from malic acid in vinegar production. On the other hand, lactic acid is produced by the fermentation of sugar and the malolactic degradation of malic acid.

### 3.3. Phenolic Compounds

The phenolic compound composition of the vinegar samples is given in Table 3. The samples were evaluated in terms of 20 different phenolic compounds. The most widely determined phenolic compounds were gallic acid, protocatechuic acid, vanillic acid, and ferulic acid in the overall vinegar samples. All compounds differed statistically across all samples (*p* < 0.05, Table 3). Guelder-rose vinegar (131.05 ± 0.44 mg/L), pomegranate vinegar (106.28 ± 0.48 mg/L), blackberry vinegar (90.89 ± 0.37 mg/L), apple vinegar (60.15 ± 0.22 mg/L), and grape vinegar (57.29 ± 0.24 mg/L) were determined to be rich in terms of phenolic content. The guelder-rose vinegar was notably high in vanillic acid (43.88 ± 0.05 mg/L), coumarin (4.77 ± 0.03 mg/L), trans-cinnamic (4.59 ± 0.05 mg/L), and gentisic acid (4.20 ± 0.06 mg/L), while the pomegranate vinegar was high in gallic acid (58.09 ± 0.18 mg/L), vanillic acid (13.64 ± 0.05 mg/L), and ascorbic acid (1.40 ± 0.01 mg/L). 

In fruit vinegars, gallic acid, protocatechuic acid, chlorogenic acid, caffeic acid, and *p*-coumaric acid were the most common phenolic chemicals [24]. Bakir et al. [31] expressed the apple vinegar phenolic compound profile as having a complex structure. Catechin, caffeic acid, syringic acid, and *p*-coumaric acid were determined in the samples. However, gallic acid and *p*-hydroxybenzoic acid were shown to be the most prevalent. In addition to this, they found that the concentration of syringic acid, caffeic acid, and *p*-coumaric acid dropped dramatically (4–10-fold) as a result of the alcoholic fermentation, whereas gallic acid and *p*-hydroxybenzoic changed significantly. Kharchoufi et al. [32] determined protocatechuic acid (28.88 ± 0.02 mg/L) and gallic acid as prominent phenolic compounds in pomegranate vinegar. Padureanu et al. [33] determined ellagic, gallic, ferulic, and chlorogenic acids in blueberry vinegar samples.

Erdal et al. [18] evaluated guelder-rose vinegar, and indicated that gallic acid was prominent, followed by ascorbic acid and protocatechuic acid. Including the peer-composition profile in this study, the phenolic compounds of traditionally produced guelder-rose vinegar samples were determined in terms of ascorbic acid, gallic acid, protocatechuic acid, hydroxybenzoic acid, vanillic acid, gentisic acid, *p*-coumaric acid, rutin, ferulic acid, *o*-coumaric acid, neohesperidin, coumarin, quercetin, trans-cinnamic acid, and flavon, respectively, as 4.61, 102.35, 2.08, 0.90, 1.78, 0.47, 0.10, 0.13, 0.22, 0.77, 0.98, 0.02, 0.14, 0.21, and 0.02 μg/mL. Compared to this study, a similar content pattern was obtained in guelder-rose vinegar, but the contents were found to be lower (Table 3). The higher content of the same phenolic compounds was supposed to result from the raw material. The use of mature and organic raw materials was a possible reason for obtaining the same phenolic compounds profile with higher amounts. As can be seen from the total acidity content, the fermentation process was found to last further and longer owing to the obtained raw material. The higher phenolic content in the samples in this study was supposed to have resulted from the raw material. The use of mature and organic raw materials was a possible reason for obtaining the same phenolic compounds profile with higher amounts.

### 3.4. The Bioactive Potential of Vinegar Samples

**Figure 4:**
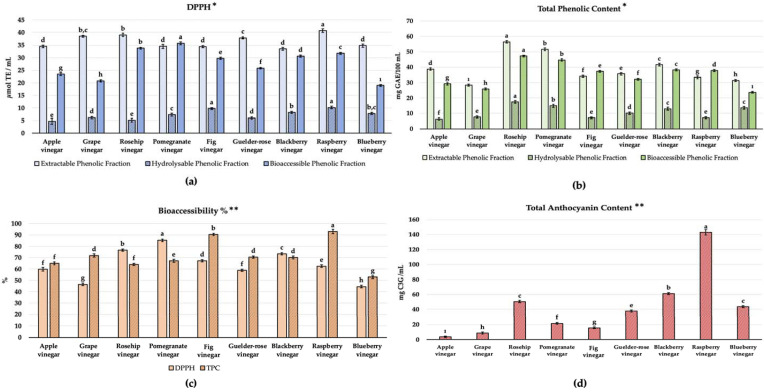
Bioactive potential of vinegar samples. (**a**) TEAC_DPPH_ (μmol TE/mL), (**b**) total phenolic content (mg GAE/100 mL), (**c**) bioaccessibility %, and (**d**) total anthocyanin content (mg C3E/mL). * Values represented by bar graphs are given as mean ± SD, letters (a–ı) represent statistical differences for different phenolic fractions between samples (*p* < 0.05); ** values represented by bar graphs are given as mean ± SD, letters (a–ı) represent statistical differences between samples (*p* < 0.05).

The bioactive potential of the vinegar samples was evaluated in terms of the antioxidant capacity (DPPH AC assay) and TPC (Folin Ciocalteu’s method) and their bioaccessibility %; the results can be seen in Figure 4a–c. Raspberry, rosehip, and pomegranate vinegar were notably higher in the TEAC_DPPH_ results; rosehip, pomegranate, and blackberry vinegar were remarkable in the TPC for EPF, HPF, and BPFs. All samples were statistically different in terms of the three different extractions (*p* < 0.05). Also, for characterizing red fruit vinegar’s bioactive potential and evaluating the effect of the anthocyanin content, the TAC results are given in Figure 4b (*p* < 0.05). Raspberry vinegar had a notably higher TAC (142.86 ± 2.50 mg C3E/L) and was followed by blackberry and rosehip vinegar (61.30 ± 0.34 mg C3E/L, 50.4 ± 0.45 mg C3E/L).

According to Liu et al. [24], the high antioxidant content and significant antioxidant activity of fruit vinegar samples can be attributed to phenolic components. Sengun et al. [21] evaluated fruit vinegar in terms of the microbiological and bioactive properties. Blackberry, rosehip, and pomegranate vinegar had the highest TPC results. Being peer-vinegar samples in this study, the TPC of apple, grape, fig, pomegranate, rosehip, and blackberry vinegar was determined as 988.0, 1025.1, 935.5, 1044.0, 1103.5, and 1162.0 mg GAE/L, respectively (in terms of EPF). Furthermore, the TEAC_DPPH_ of the same samples was 0.147, 0.119, 0.047, 0.143, 0.111, and 0.099 μg TE/mL, respectively (in terms of EPF). Although there are differences between studies in terms of the methodology, extraction method used, and expression of results in different units for spectrophotometric in vitro AC assays [34], a similar bioactive content pattern was observed among vinegar samples compared to this study. They also associated the obtained results with the content of the phenolic compound content of vinegars. Additionally, previous studies clearly showed that the AC of wines and vinegar was highly correlated with their phenolic compound content [1,33]. Gao et al. [35] assessed the black wolfberry vinegar fermentation process for 60 days. They indicated the determination of a dramatic fluctuation in the total flavonoid content and TPC during spontaneous fermentation. The TPC was found to be increased until the 15th (2.30 mg GAE/mL) day and decreased from the 20th to the 25th day to the lowest amount (2.08 mg GAE/mL). They associated changes with the macromolecular depolymerization of phenolic compounds and the conversion of individual compounds by the lactic acid bacteria strains. For the TAC of the samples, they observed a 5.63% reduction during the fermentation and concluded by the presence of diverse yeast strains with diverse abilities to adsorb anthocyanins during the process of spontaneous fermentation.

Bioactive compounds are formed in complex structures in foods. After ingestion, they are exposed to the digestive process and can manifest their potential metabolically in circulation [6]. Bioaccessibility refers to bioactive compounds that can be present and released during the process of gastrointestinal digestion and subsequently absorbed by the intestines. From a nutritional and bioactive perspective, bioaccessibility is a key indicator for food and food products. The bioaccessibility % was obtained for each assay based on the results of EPF, HPF, and BPFs, as the reflection ratio of the amount can be released by the digestion procedure. Again, raspberry vinegar has the highest TPC; pomegranate vinegar has the highest TEACDPPH among the bioaccessibility % values (Figure 4c, *p* < 0.05).

The bioaccessibility of phenolic compounds is limited by plant cell localization. For instance, blueberries have phenolic compounds in plant cell walls and vacuoles. Extractable phenolics in vacuoles are called dietary antioxidants, which are easily extractable and present in only 15% of the entire phenolics. The human gastrointestinal tract easily digests and absorbs these dietary antioxidants. However, up to 85% of plant cell wall (poly)phenols are physically entrapped in the complex matrix or covalently cross-linked with cell wall components such as cellulose, pectin, hemicellulose, and lignin. Because upper gastrointestinal tract digestive enzymes cannot access these bonded phenolic compounds, they are called non-extractable or macromolecular antioxidants [6,36].

The vinegar fermentation process can be regarded as a more advantageous value-added technique for fruits due to its ability to modify the bioactive content profile positively. The levels of phenolic compounds, anthocyanins, and organic acids undergo modifications throughout the process of fermentation. Also, the primary reason for the change in the anthocyanin content can be attributed to the biotransformation of anthocyanins into phenolic acids facilitated by the microorganisms employed during the fermentation process [6,37].

### 3.5. Chemometrics

**Figure 5:**
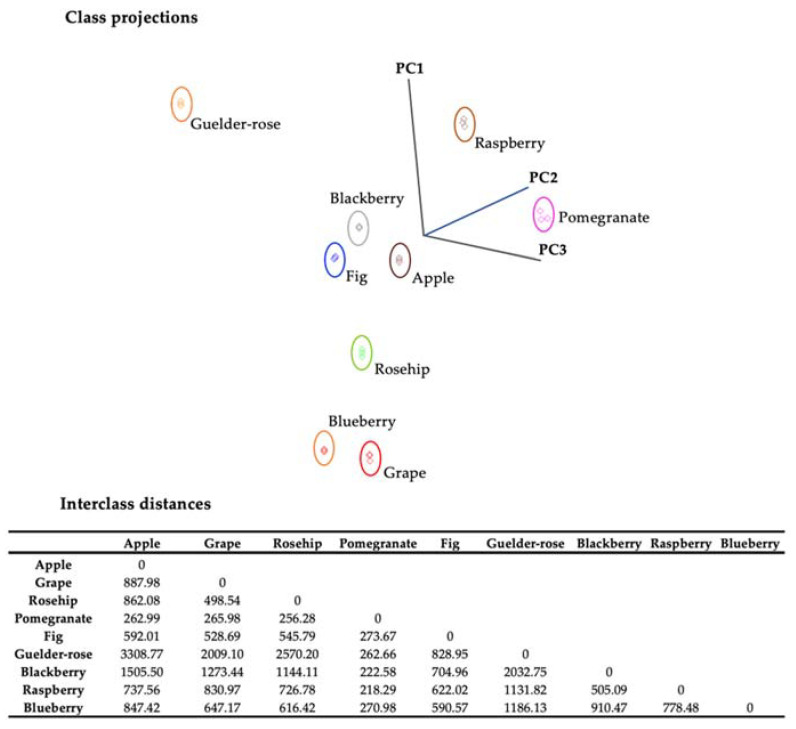
Soft independent modeling of class analogy (SIMCA) 3D projection plots of data collected by SIFT-MS for fruit vinegars. Boundaries marked around the vinegar clusters represent a 95% confidence interval. Interclass distances between vinegars were based on the SIMCA class projections.

Data interpretation can be complicated due to the large number of components. Additionally, pattern recognition was used to simultaneously assess a large number of factors by reducing them to a few fundamental qualities, creating outstanding profile discrimination. The samples identified as outliers based on the reference analysis were excluded from the dataset prior to conducting the SIMCA analysis. The vinegar samples exhibited distinct clusters when class projections were conducted using the first three principal components. Multivariate statistical analyses allow for the discrimination of fruit vinegars based on their biochemical properties. Interclass distances (ICDs) greater than 3 indicate that samples were significantly different [38]. ICD values of fruit vinegars were greater than 3 (Figure 5), demonstrating a clear discrimination of different types of vinegar based on their composition and bioactive potential.

It can be observed that all classes exhibited independence from one another. Furthermore, cross-validation analysis demonstrated a complete absence of misclassification, suggesting that the model is robust and effectively reduces overfitting. It has been observed that the most effective parameters in revealing the differences in vinegars are pH and total acidity (Table 4), which reflect the potential of vinegar fermentation on the samples. Isocitric acid is not prominent in the previously conducted vinegar studies. The phenolic compounds were afterward accompanied by the parameters related to the acidity potential. Various studies have noted vinegar samples for their gallic acid, hydroxybenzoic acid, and ferulic acid content [39,40]. In addition to supporting these findings, the related phenolic compounds were also found to be discriminative for the red fruit vinegar samples in this study. Unlike previous studies, resveratrol has been identified as a potential discriminative content among red fruit vinegars (Table 4).

dL-isocitric acid and gallic acid compounds provide separation and come to the fore due to their high content in pomegranate vinegar. Kharchoufi et al. [32] evaluated pomegranate juice, wine, and vinegar. They observed the increase in the gallic and protocatechic acid contents, respectively, in juice, wine, and vinegar. Also, this increase is attributed to the hydrolysis of hydrolyzable tannins. Conversely, the levels of tyrosol and catechin experienced a modest decline from the wine stage to the vinegar stage. Being the most widely detected phenolic compounds in fruit vinegar samples, Liu et al. [24] correlated the presence of protocatechuic acid, caffeic acid, gallic acid, chlorogenic acid, and *p*-coumaric acid with the prevention of damage caused by oxidative stress. Furthermore, rosehip vinegar (14.77 ± 0.01 mg/L) and blackberry vinegar (10.67 ± 0.04 mg/L) have come to the fore due to their high protocatechuic acid content among the vinegar samples.

Ousaaid et al. [40] evaluated the antianemic properties of apple vinegar. They determined trans-ferulic acid, ferulic acid, and sinapic acid as the most abundant bioactive compounds and associated with the antioxidative, anti-inflammatory, and antianemic properties of the vinegar samples. In this study, apple vinegar had the highest content of ferulic acid with 24.61 ± 0.05 mg/L and the closest sample was pomegranate vinegar with 20.49 ± 0.04 mg/L ferulic acid content.

### 3.6. Linear Correlation

The linear correlations among the obtained results between the vinegar samples are given in Supplementary Tables S1 and S2. There are significant positive and negative correlations identified for organic acids, phenolic compounds, and AC assay parameters. In terms of the AC assays, remarkably, there are positive correlations (97.42%) between the BPF results of the DPPH and TPC in evaluating the bioactive potential (Supplementary Table S2). Additionally, the EPF and BPF of the TPC had an 84.58% correlation (Supplementary Table S2). This indicates that the bioactive potential of the samples correlated via the phenolic fractions regarding the AC assays. Liu et al. [22] found a strong positive correlation between the AC assays (R^2^ = 0.971–0.990) and TPC as an indication of the presence of phenolic components provided to the oxidation-reducing and radical removal potential in fruit vinegars.

For organic acids, oxalic acid-dihydrate, oxalic acid, and butyric acid had the most, above 90%, in relation to the determined organic acids due to the phenolic compounds. For the phenolic compounds, there was, respectively, a 91.26%, 91.85%, and 92.57% relationship between the quercetin-*p-*coumaric acid, quercetin-hesperidin, and hesperidin-ferulic acid compound couples (Supplementary Table S1). Es-sbata et al. [41] evaluated cactus plant vinegar production, focusing on phenolic and volatile compounds, particularly emphasizing the acidification process. The researchers identified a correlation among the compounds belonging to the tyrosol, hesperidin, naringenin, protocatechualdehyde, and ferulic acid groups, as well as among the compounds belonging to the catechin, *p*-coumaric acid, ferulic acid, and *p*-hydroxybenzoic acid groups.

## 4. Conclusions

The process of vinegar fermentation is a highly beneficial bioprocess that enhances the nutritional and bioactive characteristics of food products. The organic acid and phenolic compound content due to the bioactive potential of rosehip, pomegranate, fig, guelder-rose, blackberry, raspberry, blueberry, grape, and apple vinegars were evaluated in this study. The most widely determined organic acids were acetic acid, citric acid, succinic acid, malic acid, adipic acid, and lactic acid, while the phenolic compounds were gallic acid, protocatechuic acid, vanillic acid, and ferulic acid in the vinegar samples. Raspberry, pomegranate, and rosehip vinegar were determined to have a notable bioactive potential (DPPH antioxidant capacity assay, total phenolic content); raspberry vinegar, especially, showed greater bioaccessible properties. As a result of the conducted analyses and data evaluation, the pH and total acidity were the most effective discriminative parameters for the samples. The higher acidic potential of the vinegar indicates an advanced fermentation process. Utilizing the organic and mature raw material with a higher sugar and phenolic compound content is thought to be the core reason for the proper fermentation. For chemometric analyses, the acidity parameters were followed by the phenolic compounds and organic acids regarding the discriminating power. Gallic acid, hydroxybenzoic acid, ascorbic acid, ferulic acid, and resveratrol were discriminative for the phenolic compounds; dL-isocitric acid, acetic acid, and succinic acid were discriminative regarding the organic acids. Additionally, the dL-isocitric acid and resveratrol determination in the red fruit vinegar samples were included as outstanding outputs. Therefore, vinegar production from red fruits could be considered an excellent method not only to increase the shelf life and the shelf stability but also to convert the macromolecular antioxidants to dietary antioxidants, facilitating their utilization within the body and promoting health-related attributes.

## Figures and Tables

**Table 1:** Performance parameters of analytical method for organic acids methodology (Wavelength: 210 nm).

Organic Acid	Cas Number	Linearity Calibration Curve (mg/L)	Regression Equation *	Correlation Coefficient	LOD (µg/kg)	LOQ (µg/kg)
**Maleic acid**	110-16-7	0.50–5.0 0	y = 89.3899x + 0.2609	0.9999	16.65	55.07
**Malonic acid**	141-82-2	100.00–1000.00	y = 0.7908x + 1.5608	0.9999	102.55	341.85
**Barbituric acid**	67-52-7	0.50–5.00	y = 70.1854x − 0.8500	0.9998	1.35	4.50
**Phytic acid**	83-86-3	400.00–4000.00	y = 0.1575x − 28.0443	0.9962	162.11	540.36
**DL-Isocitric acid**	1637-73-6	16.00–160.0	y = 4.8577x − 1.9689	0.9999	14.75	49.16
**(-)Quinic acid**	77-95-2	200.00–2000.00	y = 0.4157x + 0.5559	0.9999	83.24	277.47
**Shikimic acid**	138-59-0	2.50–25.00	y = 47.4045x + 1.5608	0.9999	1.10	3.66
**Adipic acid**	124-04-9	200.00–2000.00	y = 0.5847x − 1.1275	0.9998	131.47	438.24
**Oxalic-Dihydrate**	6153-56-6	8.00–80.00	y = 10.9688x + 0.0661	0.9999	7.29	24.30
**Oxalic acid**	144-62-7	12.00–60.00	y = 12.7195x − 0.6878	0.9999	6.39	21.29
**Citric acid**	77-92-9	80.00–400.00	y = 0.8280x + 0.0829	0.9998	3.96	13.20
**D-(−)-Tartaric acid**	147-71-7	60.00–300.00	y = 1.0772x + 1.5076	0.9999	14.37	47.90
**D-(+)-Malic acid**	636-61-3	96.00 –480.00	y = 0.7336x − 0.1788	0.9997	16.77	55.90
**Succinic acid**	110-15-6	112.00–560.00	y = 0.4397 x + 2.2500	0.9997	70.11	233.70
**L-(+)-Lactic acid**	79-33-4	120.00–600.00	y = 0.5787 x + 1.2572	0.9999	43.86	146.20
**Formic acid**	64-18-6	20.00–100.00	y = 3.9434 x − 2.6180	0.9994	22.05	73.50
**Acetic acid**	64-19-7	180.00–900.00	y = 0.2598x − 0.0077	0.9998	30.39	101.30
**Fumaric acid**	110-17-8	1.00–5.00	y = 112.0394x + 0.4167	0.9998	18.48	61.60
**Propionic acid**	79-09-4	40.00–200.00	y = 3.7163x + 8.8626	0.9997	26.16	87.02
**Isobutyric acid**	79-31-2	40.00–200.00	y = 4.5562x + 0.6342	0.9998	25.59	85.30
**Butyric acid**	107-92-6	60.00–200.00	y = 3.1371 x + 1.0894	0.9999	32.86	109.54

* x: Concentration of the analytical standard; y: Response.

**Table 2:** Organic acid content of vinegar samples.

Organic Acid (mg/L)	Apple Vinegar	Grape Vinegar	Rosehip Vinegar	Pomegranate Vinegar	Fig Vinegar	Guelder-Rose Vinegar	Blackberry Vinegar	Raspberry Vinegar	Blueberry Vinegar
**Maleic Acid**	nd. *	nd.	nd.	nd.	nd.	0.93 ± 0.00 ^b^	0.23 ± 0.01 ^c^	1.25 ± 0.01 ^a^	nd.
**Malonic acid**	31.59 ± 0.06 ^d^ **	6.53 ± 0.18 ^f^	64.43 ± 0.08 ^c^	nd.	13.73 ± 0.13 ^e^	74.24 ± 0.17 ^b^	nd.	nd.	123.01 ± 0.15 ^a^
**Barbituric acid**	3.38 ± 0.11 ^c^	3.14 ± 0.09 ^d^	4.59 ± 0.03 ^b^	4.66 ± 0.34 ^b^	1.15 ± 0.03 ^e^	11.93 ± 0.06 ^a^	4.68 ± 0.04 ^b^	2.01 ± 0.02 ^e^	3.20 ± 0.07 ^d^
**Oxalic-Dihydrate**	6.27 ± 0.03 ^a^	nd.	nd.	nd.	0.86 ± 0.00 ^b^	nd.	nd.	nd.	nd.
**DL-Isocitric acid**	4.50 ± 0.05 ^ı^	16.05 ± 0.09 ^f^	40.44 ± 0.21 ^d^	967.35 ± 1.07 ^a^	26.16 ± 0.08 ^e^	6.78 ± 0.15 ^g^	155.95 ± 0.10 ^c^	325.49 ± 0.30 ^b^	5.17 ± 0.07 ^h^
**(-)Quinic acid**	nd.	nd.	nd.	nd.	nd.	143.63 ± 0.22 ^b^	nd.	nd.	244.75 ± 0.82 ^a^
**Shikimic acid**	4.21 ± 0.21 ^f^	4.60 ± 0.14 ^e^	6.55 ± 0.12 ^c^	7.39 ± 0.19 ^b^	1.55 ± 0.03 ^h^	17.67 ± 0.02 ^a^	6.86 ± 0.07 ^d^	2.87 ± 0.08 ^g^	4.58 ± 0.26 ^e^
**Adipic acid**	933.53 ± 0.96 ^b^	951.10 ± 0.10 ^a^	749.28 ± 0.07 ^c^	nd.	587.83 ± 0.54 ^d^	270.93 ± 0.35 ^f^	273.63 ± 0.52 ^e^	nd.	nd.
**Oxalic acid**	4.03 ± 0.10 ^a^	nd.	nd.	nd.	0.75 ± 0.00 ^b^	nd.	nd.	nd.	nd.
**Citric acid**	23.37 ± 0.59 ^g^	92.41 ± 0.52 ^f^	239.57 ± 0.38 ^d^	5335.26 ± 42.07 ^a^	160.43 ± 0.29 ^e^	36.59 ± 0.36 ^g^	903.97 ± 0.21 ^c^	1904.66 ± 1.75 ^b^	26.80 ± 0.17 ^g^
**D-(** **−)-Tartaric acid**	27.94 ± 0.21 ^c^	601.06 ± 0.90 ^a^	nd.	nd.	nd.	23.60 ± 0.16 ^d^	nd.	115.08 ± 0.05 ^b^	22.30 ± 0.15 ^e^
**D-(+)-Malic acid**	31.48 ± 0.39 ^d, e^	nd.	72.28 ± 0.15 ^c^	3971.76 ± 39.36 ^a^	23.92 ± 0.05 ^e^	55.37 ± 0.42 ^c^	626.62 ± 0.42 ^b^	51.29 ± 0.36 ^c, d^	19.72 ± 0.39 ^e, f^
**Succinic acid**	462.09 ± 0.72 ^g^	498.58 ± 1.18 ^f^	691.80 ± 0.43 ^d^	763.36 ± 1.38 ^b^	166.69 ± 0.27 ^ı^	1902.47 ± 1.67 ^a^	741.21 ± 0.64 ^c^	304.51 ± 2.69 ^h^	514.41 ± 0.49 ^e^
**L-(+)-Lactic acid**	39.62 ± 0.28 ^g^	843.07 ± 0.13 ^b^	365.26 ± 0.50 ^c^	nd.	1344.70 ± 9.27 ^a^	67.66 ± 0.17 ^e^	53.88 ± 0.26 ^f^	22.47 ± 0.08 ^h^	278.22 ± 2.40 ^d^
**Acetic acid**	48,650.63 ± 162.31 ^d^	29,968.43 ± 320.06 ^h^	39,177.90 ± 349.65 ^g^	45,912.03 ± 385.67 ^f^	47,618.77 ± 142.46 ^e^	58,806.27 ± 197.60 ^b^	49,515.90 ± 54.99 ^c^	60,592.87 ± 318.75 ^a^	30,150.70 ± 123.12 ^h^
**Fumaric acid**	0.41 ± 0.00 ^b^	0.28 ± 0.00 ^c^	4.81 ± 0.06 ^a^	nd.	nd.	nd.	nd.	nd.	nd.
**Propionic acid**	148.31 ± 1.23 ^a^	140.81 ± 0.15 ^b^	115.09 ± 0.33 ^c^	nd.	89.97 ± 0.25 ^d^	nd.	14.59 ± 0.23 ^e^	10.86 ± 0.05 ^f^	nd.
**Isobutyric acid**	nd.	nd.	nd.	nd.	nd.	nd.	nd.	13.57 ± 0.25 ^a^	nd.
**Butyric acid**	0.49 ± 0.02 ^a^	nd.	nd.	nd.	nd.	nd.	nd.	nd.	nd.
**Total**	**50,371.86** ± 35.36	**33,126.06** ± 69.81	**41,532.01** ± 79.27	**56,961.83** ± 84.13	**50,036.51** ± 31.03	**61,418.06** ± 43.08	**52,297.52** ± 11.98	**63,346.93** ± 69.50	**31,392.87** ± 29.82

* nd: Not detected. ** nd: Values are given as mean ± SD and statistical differences between vinegar samples are represented with different letters (*p* < 0.05).

**Table 3:** Phenolic compound content of vinegar samples.

Compound (mg/L)	Apple Vinegar	Grape Vinegar	Rosehip Vinegar	Pomegranate Vinegar	Fig Vinegar	Guelder-Rose Vinegar	Blackberry Vinegar	Raspberry Vinegar	Blueberry Vinegar
**Ascorbic acid**	0.13 ± 0.00 ^e^ *	1.03 ± 0.00 ^b^	0.37 ± 0.00 ^c^	1.40 ± 0.01 ^a^	0.04 ± 0.00 ^g^	nd.	0.26 ± 0.00 ^d^	0.09 ± 0.00 ^f^	nd.
**Gallic acid**	9.97 ± 0.14 ^ı^	29.21 ± 0.08 ^d^	15.75 ± 0.02 ^h^	58.09 ± 0.18 ^a^	25.87 ± 0.04 ^e^	47.98 ± 0.06 ^c^	55.44 ± 0.05 ^b^	19.65 ± 0.02 ^f^	12.75 ± 0.04 ^g^
**Protocatechuic acid**	5.52 ± 0.03 ^c^	1.26 ± 0.00 ^ı^	14.77 ± 0.01 ^a^	2.55 ± 0.02 ^e^	2.31 ± 0.02 ^g^	3.47 ± 0.01 ^d^	10.67 ± 0.04 ^b^	2.36 ± 0.01 ^f^	2.03 ± 0.00 ^h^
**Catechin**	0.28 ± 0.01 ^e^	nd.	0.28 ± 0.00 ^e^	0.81 ± 0.01 ^d^	9.10 ± 0.06 ^a^	4.92 ± 0.01 ^b^	1.36 ± 0.02 ^c^	0.09 ± 0.00 ^f^	nd.
**Hydroxybenzoic acid**	3.02 ± 0.03 ^d^	0.76 ± 0.00 ^f^	2.98 ± 0.02 ^d^	13.64 ± 0.05 ^a^	0.98 ± 0.03 ^g^	1.40 ± 0.03 ^e^	7.02 ± 0.06 ^b^	5.20 ± 0.03 ^c^	nd.
**Vanillic acid**	6.12 ± 0.02 ^c^	2.38 ± 0.00 ^g^	4.08 ± 0.04 ^e^	3.80 ± 0.03 ^f^	4.24 ± 0.02 ^d^	43.88 ± 0.05 ^a^	1.26 ± 0.06 ^h^	0.75 ± 0.00 ^ı^	9.98 ± 0.08 ^b^
**Gentisic acid**	nd.	nd.	1.48 ± 0.01 ^c^	nd.	1.75 ± 0.02 ^b^	4.20 ± 0.06 ^a^	0.44 ± 0.01 ^d^	0.27 ± 0.01 ^e^	nd.
***p*-coumaric acid**	4.99 ± 0.03 ^a^	0.09 ± 0.00 ^f^	0.10 ± 0.00 ^f^	0.09 ± 0.00 ^f^	0.17 ± 0.00 ^e^	0.70 ± 0.00 ^b^	0.16 ± 0.00 ^e^	0.31 ± 0.01 ^c^	0.23 ± 0.00 ^d^
**Rutin**	5.52 ± 0.06 ^a^	2.22 ± 0.01 ^d^	2.20 ± 0.03 ^d, e^	2.16 ± 0.02 ^e^	2.71 ± 0.00 ^b^	1.90 ± 0.00 ^f^	0.91 ± 0.01 ^h^	1.54 ± 0.05 ^g^	2.44 ± 0.00 ^c^
**Ferulic acid**	24.61 ± 0.05 ^a^	0.74 ± 0.03 ^f^	2.73 ± 0.03 ^e^	20.49 ± 0.04 ^b^	0.25 ± 0.01 ^g^	0.75 ± 0.03 ^f^	8.68 ± 0.01 ^c^	5.99 ± 0.02 ^d^	0.75 ± 0.00 ^f^
**Naringin**	nd.	15.79 ± 0.06 ^a^	2.72 ± 0.05 ^d^	0.71 ± 0.00 ^h^	7.15 ± 0.04 ^b^	6.89 ± 0.03 ^c^	1.23 ± 0.01 ^f^	1.13 ± 0.00 ^g^	2.37 ± 0.02 ^e^
***o*-coumaric acid**	0.56 ± 0.01 ^ı^	2.36 ± 0.03 ^c^	5.54 ± 0.02 ^a^	0.99 ± 0.03 ^g^	0.74 ± 0.04 ^h^	3.33 ± 0.02 ^b^	1.97 ± 0.04 ^d^	1.12 ± 0.01 ^f^	1.62 ± 0.00 ^e^
**Neohesperidin**	0.66 ± 0.02 ^b^	0.77 ± 0.01 ^a^	nd.	0.30 ± 0.01 ^f^	0.59 ± 0.03 ^c^	0.35 ± 0.02 ^e^	0.44 ± 0.04 ^d^	0.60 ± 0.03 ^c^	0.21 ± 0.04 ^g^
**Coumarin**	1.15 ± 0.03 ^c^	0.08 ± 0.00 ^f^	1.52 ± 0.01 ^b^	0.13 ± 0.00 ^e^	0.15 ± 0.01 ^e^	4.77 ± 0.03 ^a^	0.15 ± 0.00 ^e^	0.29 ± 0.01 ^d^	0.05 ± 0.00 ^f^
**Resveratrol**	nd.	0.03 ± 0.00 ^h^	0.07 ± 0.00 ^f^	0.34 ± 0.01 ^d^	0.86 ± 0.02 ^a^	0.41 ± 0.00 ^c^	0.10 ± 0.00 ^e^	0.05 ± 0.00 ^g^	0.48 ± 0.01 ^b^
**Quercetin**	1.63 ± 0.02 ^b^	0.38 ± 0.00 ^f^	0.38 ± 0.01 ^f^	0.36 ± 0.04 ^f, g^	0.33 ± 0.01 ^g^	1.11 ± 0.03 ^d^	0.46 ± 0.01 ^e^	1.34 ± 0.01 ^c^	1.76 ± 0.02 ^a^
**Trans-cinnamic**	1.33 ± 0.02 ^c^	0.17 ± 0.00 ^f^	0.09 ± 0.00 ^g^	0.10 ± 0.00 ^g^	0.12 ± 0.01 ^g^	4.59 ± 0.05 ^a^	0.34 ± 0.01 ^e^	0.74 ± 0.04 ^d^	2.01 ± 0.02 ^b^
**Hesperidin**	1.57 ± 0.06 ^a^	nd.	0.14 ± 0.00 ^d^	nd.	0.03 ± 0.00 ^e^	0.40 ± 0.01 ^b^	nd.	nd.	0.19 ± 0.01 ^c^
**Alizarin**	nd.	0.03 ± 0.00 ^c^	nd.	0.34 ± 0.02 ^a^	0.10 ± 0.00 ^b^	nd.	nd.	nd.	nd.
**Total**	**60.15** ± 0.22	**57.29** ± 0.24	**55.21** ± 0.26	**106.28** ± 0.48	**57.49** ± 0.36	**131.05** ± 0.44	**90.89** ± 0.37	**41.53** ± 0.24	**36.86** ± 0.24

* Values are given as mean ± SD and statistical differences between samples are represented with different letters (*p* < 0.05).

**Table 4:** Discriminating power of the parameters belonging to vinegar samples.

Parameter	Discriminating Power (10^4^)
**pH**	1219
**Total acidity**	761
**dL-isocitric acid**	413
**Gallic acid**	366
**Hydroxybenzoic acid**	180
**Barbituric acid**	125
**Protocatechuic acid**	76
**Ascorbic acid**	71
**Acetic acid**	56
**Adipic acid**	30
***o*-coumaric acid**	26
**Ferulic acid**	24
**Resveratrol**	14
**Succinic acid**	11

## Data Availability

Data will be available from the corresponding author upon reasonable request.

## Supplementary Materials

The following supporting information can be downloaded at: https://www.mdpi.com/article/10.3390/foods12203769/s1, Table S1: linear relationships between pH, total acidity, organic acid, and phenolic compounds of vinegar samples; Table S2: linear relationships between total anthocyanin content, antioxidant capacity (DPPH), and total phenolic content.
