# Supplementary material for: Characterization of Fruit Vinegars via Bioactive and Organic Acid Profile Using Chemometrics
Source: Foods. 2023 Oct 13;12(20):3769. doi: 10.3390/foods12203769 (PMC10606018; doi:10.3390/foods12203769)
Supplement: Supplementary file 1 [file foods-12-03769-s001.zip › foods-2608970-supplementary.pdf]

Supp. Table S1. Linear relationships between pH, total acidity, organic acid and phenolic compounds of vinegar samples

	1	2	3	4	5	6	7	8	9	10	11	12	13	14	15	16	17	18	19	20	21	22	23	24	25	26	27	28	29	30	31	32	33	34	35	36	37	38	39	40
**1***	1.00																																							
**2**	-0.34	1.00																																						
**3**	-0.68	0.55	1.00																																					
**4**	0.24	-0.60	-0.09	1.00																																				
**5**	-0.10	-0.11	0.36	0.34	1.00																																			
**6**	0.00	0.06	-0.24	-0.05	-0.16	1.00																																		
**7**	-0.13	0.42	0.03	-0.45	-0.05	-0.22	1.00																																	
**8**	-0.12	-0.52	0.08	0.88	0.37	-0.21	-0.29	1.00																																
**9**	-0.10	-0.10	0.36	0.32	0.99	-0.22	0.00	0.37	1.00																															
**10**	0.49	-0.32	-0.46	-0.14	-0.15	0.51	-0.54	-0.44	-0.19	1.00																														
**11**	0.02	0.07	-0.25	-0.06	-0.18	0.99	-0.23	-0.22	-0.23	0.52	1.00																													
**12**	-0.14	0.44	0.04	-0.46	-0.06	-0.23	1.00	-0.29	-0.01	-0.54	-0.24	1.00																												
**13**	-0.08	-0.42	-0.06	-0.27	-0.17	-0.14	-0.18	-0.18	-0.16	0.44	-0.15	-0.18	1.00																											
**14**	0.04	0.23	-0.21	-0.35	0.06	-0.17	0.95	-0.21	0.11	-0.42	-0.17	0.94	-0.20	1.00																										
**15**	-0.11	-0.12	0.36	0.34	0.99	-0.21	-0.02	0.39	0.99	-0.18	-0.23	-0.03	-0.16	0.08	1.00																									
**16**	0.53	-0.16	-0.40	-0.11	-0.43	-0.13	-0.37	-0.16	-0.43	0.46	-0.09	-0.37	0.35	-0.31	-0.42	1.00																								
**17**	-0.50	0.86	0.76	-0.35	0.34	0.12	0.19	-0.24	0.33	-0.32	0.12	0.20	-0.45	0.04	0.33	-0.38	1.00																							
**18**	0.68	-0.19	-0.24	0.24	0.02	-0.07	-0.19	-0.21	0.00	0.39	-0.08	-0.19	-0.12	-0.16	-0.01	0.03	-0.25	1.00																						
**19**	0.51	-0.27	-0.49	-0.17	-0.35	0.55	-0.45	-0.49	-0.39	0.97	0.56	-0.45	0.44	-0.38	-0.39	0.48	-0.37	0.41	1.00																					
**20**	-0.59	0.61	0.77	-0.30	-0.28	-0.14	0.18	-0.18	-0.27	-0.40	-0.15	0.20	0.05	-0.14	-0.27	-0.25	0.52	-0.14	-0.27	1.00																				
**21**	-0.05	0.02	-0.21	-0.03	-0.11	0.99	-0.20	-0.18	-0.16	0.49	0.98	-0.20	-0.11	-0.15	-0.16	-0.24	0.10	-0.05	0.52	-0.12	1.00																			
**22**	0.19	-0.14	-0.41	-0.47	-0.09	-0.08	0.68	-0.43	-0.05	0.12	-0.10	0.67	0.43	0.75	-0.07	-0.05	-0.34	0.01	0.16	-0.23	-0.05	1.00																		
**23**	-0.27	0.25	0.07	-0.44	0.50	-0.18	0.54	-0.20	0.53	-0.28	-0.18	0.54	-0.13	0.65	0.52	-0.32	0.36	-0.38	-0.42	-0.27	-0.16	0.43	1.00																	
**24**	-0.10	0.14	-0.22	-0.18	-0.11	0.92	0.12	-0.29	-0.15	0.32	0.91	0.11	-0.19	0.17	-0.15	-0.39	0.17	-0.09	0.38	-0.10	0.95	0.17	0.06	1.00																
**25**	0.28	-0.01	-0.27	0.04	0.05	0.51	-0.25	-0.33	0.00	0.46	0.49	-0.25	-0.36	-0.17	0.00	-0.31	0.05	0.64	0.43	-0.24	0.54	-0.13	-0.08	0.51	1.00															
**26**	-0.19	0.47	-0.07	-0.53	-0.04	0.23	0.83	-0.47	-0.01	-0.26	0.22	0.83	-0.31	0.82	-0.03	-0.56	0.32	-0.12	-0.20	0.07	0.27	0.58	0.59	0.56	0.23	1.00														
**27**	-0.10	-0.11	0.36	0.49	0.87	0.14	-0.28	0.52	0.84	-0.03	0.13	-0.29	-0.20	-0.19	0.85	-0.26	0.36	-0.15	-0.21	-0.24	0.16	-0.34	0.21	0.05	-0.04	-0.24	1.00													
**28**	0.16	0.12	0.40	0.30	0.76	-0.21	-0.31	0.25	0.75	0.00	-0.20	-0.31	-0.27	-0.25	0.75	0.09	0.45	0.12	-0.18	-0.17	-0.24	-0.43	0.20	-0.35	-0.04	-0.38	0.79	1.00												
**29**	-0.07	0.03	-0.18	-0.01	-0.08	0.99	-0.21	-0.16	-0.13	0.47	0.98	-0.21	-0.12	-0.16	-0.12	-0.25	0.13	-0.06	0.51	-0.12	1.00	-0.07	-0.15	0.94	0.53	0.25	0.20	-0.20	1.00											
**30**	0.49	-0.36	-0.03	0.40	0.46	-0.23	-0.35	0.05	0.45	0.34	-0.24	-0.35	0.01	-0.28	0.45	-0.09	-0.17	0.85	0.24	-0.25	-0.18	-0.08	-0.17	-0.27	0.51	-0.30	0.25	0.44	-0.17	1.00										
**31**	-0.28	-0.12	0.34	0.52	0.67	0.43	-0.36	0.55	0.63	-0.01	0.41	-0.37	-0.22	-0.30	0.64	-0.40	0.34	-0.25	-0.13	-0.12	0.46	-0.44	0.03	0.34	0.07	-0.18	0.91	0.51	0.50	0.07	1.00									
**32**	0.25	0.33	0.03	-0.07	0.11	-0.03	-0.21	-0.02	0.12	0.07	0.01	-0.21	-0.28	-0.15	0.12	0.61	0.33	-0.23	-0.01	-0.22	-0.15	-0.37	0.15	-0.24	-0.24	-0.30	0.31	0.63	-0.13	-0.20	0.11	1.00								
**33**	0.04	-0.03	-0.27	0.02	-0.14	0.99	-0.21	-0.15	-0.19	0.52	0.99	-0.22	-0.10	-0.15	-0.19	-0.15	0.04	-0.03	0.57	-0.17	0.99	-0.03	-0.22	0.92	0.49	0.22	0.16	-0.23	0.99	-0.18	0.45	-0.10	1.00							
**34**	0.20	-0.44	-0.11	-0.08	0.06	-0.29	-0.37	-0.02	0.07	0.47	-0.27	-0.37	0.82	-0.30	0.08	0.66	-0.39	-0.09	0.39	-0.23	-0.31	0.21	-0.06	-0.46	-0.46	-0.59	0.09	0.21	-0.31	0.14	-0.10	0.23	-0.27	1.00						
**35**	-0.27	0.12	-0.07	-0.33	-0.25	0.88	-0.21	-0.33	-0.29	0.52	0.88	-0.21	0.22	-0.21	-0.29	-0.01	0.16	-0.35	0.55	0.05	0.87	0.01	-0.09	0.80	0.22	0.17	0.04	-0.30	0.87	-0.44	0.33	-0.02	0.86	0.00	1.00					
**36**	-0.01	0.01	0.33	0.35	0.79	0.37	-0.32	0.22	0.76	0.21	0.36	-0.33	-0.23	-0.25	0.76	-0.33	0.45	0.11	0.06	-0.18	0.40	-0.31	0.13	0.29	0.31	-0.11	0.91	0.73	0.43	0.41	0.86	0.20	0.39	-0.03	0.22	1.00				
**37**	0.31	0.07	-0.15	0.25	-0.01	-0.23	-0.02	0.37	0.01	-0.27	-0.20	-0.03	-0.35	0.06	0.01	0.55	0.00	-0.29	-0.29	-0.27	-0.34	-0.28	0.02	-0.38	-0.50	-0.30	0.19	0.40	-0.33	-0.32	0.03	0.81	-0.26	0.12	-0.30	-0.08	1.00			
**38**	-0.27	-0.04	0.02	0.25	-0.05	0.88	-0.27	0.17	-0.10	0.21	0.86	-0.27	-0.17	-0.27	-0.09	-0.38	0.13	-0.17	0.26	0.06	0.90	-0.28	-0.31	0.81	0.36	0.10	0.29	-0.21	0.91	-0.22	0.64	-0.23	0.89	-0.39	0.76	0.42	-0.28	1.00		
**39**	-0.04	-0.01	-0.18	0.05	-0.03	0.98	-0.24	-0.11	-0.08	0.48	0.97	-0.24	-0.14	-0.18	-0.07	-0.26	0.12	-0.03	0.50	-0.15	0.99	-0.10	-0.16	0.93	0.54	0.22	0.25	-0.16	0.99	-0.13	0.54	-0.12	0.99	-0.31	0.84	0.48	-0.31	0.92	1.00	
**40**	0.20	0.25	-0.29	-0.37	-0.08	-0.15	0.88	-0.25	-0.03	-0.31	-0.14	0.87	-0.14	0.94	-0.05	-0.01	-0.01	-0.19	-0.24	-0.17	-0.17	0.73	0.52	0.10	-0.32	0.68	-0.21	-0.20	-0.19	-0.38	-0.36	0.09	-0.14	-0.13	-0.17	-0.30	0.30	-0.33	-0.21	1.00

*1: Total acidity; 2: pH; 3: Maleic acid; 4: Malonic acid; 5: Barbituric acid; 6: Oksalic-dyhidrate 7: DL-isocitric acid; 8: (-)Quinic acid; 9: Shikimic acid; 10: Adipic acid; 11: Oxalic acid; 12: Citric acid; 13: D-(−)-Tartaric acid; 14: D-(+)-Malic acid; 15:Succinic acid; 16: L-(+)-Lactic acid; 17: Acetic acid; 18: Fumaric acid; 19: Propionic acid; 20: Isobutyric acid; 21: Butyric acid; 22: Ascorbic acid; 23: Gallic acid; 24: Ferulic acid; 25: Protocatechuic acid; 26: Hydroxybenzoic acid; 27: Vanillic acid; 28: Gentisic acid; 29: *p*-coumaric acid; 30: *o*-coumaric acid; 31: Trans-sinamic acid; 32: Catechin; 33: Rutin; 34: Naringin; 35: Neohesperidin; 36: Coumarin; 37: Resveratrol; 38: Quarcetin; 39: Hesperidin; 40: Alizarin

foods

MDPI

Supp. Table S2. Linear relationships between total anthocyanin content, antioxidant capacity (DPPH) and Total phenolic content

	TAC	DPPH-E	DPPH-H	DPPH-B	DPPH-B%	TPC-E	TPC-H	TPC-B	TPC-B%
**TAC***	1.00								
**DPPH-E**	0.60	1.00							
**DPPH-H**	0.55	0.00	1.00						
**DPPH-B**	0.35	0.17	0.26	1.00					
**DPPH-B%**	0.07	-0.11	0.06	**0.94**	1.00				
**TPC-E**	-0.04	0.00	-0.34	**0.75**	**0.85**	1.00			
**TPC-H**	0.02	-0.05	-0.23	0.40	0.48	**0.75**	1.00		
**TPC-B**	0.26	0.18	0.11	**0.97**	**0.94**	**0.85**	0.52	1.00	
**TPC-B%**	0.44	0.34	0.67	0.39	0.16	-0.27	-0.59	0.26	1.00

*TAC: Total anthocyanin content; DPPH: Trolox equivalent antioxidant capacity according to DPPH method; TPC: Total phenolic content; E: Extractable phenolic fraction; H: Hydrolysable phenolic fraction; B: Bioaccessible phenolic fraction; B% Bioaccessibility
